# Long‐term fertilization and cultivation impacts on nematode abundance and community structure in tall fescue turfgrass

**DOI:** 10.1002/ece3.10905

**Published:** 2024-02-09

**Authors:** Benjamin D. Waldo, Fereshteh Shahoveisi, Mark J. Carroll

**Affiliations:** ^1^ Mycology and Nematology Genetic Diversity and Biology Laboratory USDA‐ARS Beltsville Maryland USA; ^2^ Department of Plant Sciences and Landscape Architecture University of Maryland College Park Maryland USA

**Keywords:** enhanced efficiency fertilizer, long‐term turfgrass management, nematode community, soil food web

## Abstract

Impacts of long‐term fertilization and cultivation were evaluated on nematode communities associated with tall fescue turfgrass following 11 years of treatment applications. Fertilizer treatments of biosolid, synthetic, and plant‐based fertilizers and cultivation treatments of 0×, 1×, and 2× aerification passes were applied to randomized and replicated tall fescue plots at the University of Maryland Paint Branch Turfgrass facility in College Park, Maryland. Free‐living and plant‐parasitic nematodes were identified, enumerated, and categorized into functional groups. Nematode count data were compared using generalized linear mixed modeling with negative binomial distribution and two‐way ANOVA was used to compare nematode ecological indices. Biosolid treatments resulted in lower omnivore‐predator densities than plant‐based fertilizer treatments (*p* ≤ .001) and significantly greater *Hoplolaimus* densities than plant‐based fertilizer plots (*p* ≤ .05). Synthetic fertilizer applications resulted in the greatest *Eucephalobus* (*p* ≤ .05) and total bacterivore densities (*p* ≤ .001) of all fertilizer treatments. Plant‐based fertilizer‐treated plots had the largest Maturity Index cp 2‐5 and Structure Index (*p* ≤ .05). Cultivation of 1× resulted in fewer total bacterivore densities than 2× (*p* ≤ .01) while omnivore‐predator densities were greater in 1× than 0× (*p* ≤ .001). Plant health, as measured by NDVI, was lowest in biosolid‐treated turfgrass (*p* ≤ .05). These findings suggest that long‐term turfgrass management practices can have variable impacts on nematode abundance and community structure in tall fescue and provide insights into ecological impacts of turfgrass management practices.

## Graphical abstract

Turfgrass fertilizer applications over 11 years had variable effects on soil nematode abundance and community structure. Biosolid‐based fertilizer applications where Cu, Fe, and Zn concentrations were highest resulted in the highest plant‐parasitic *Hoplolaimus* abundance and lowest beneficial omnivore‐predator nematode abundance.

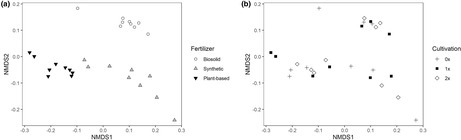

## 1 INTRODUCTION

Nematodes are important members of the soil food web and are abundant in soil environments. These microscopic roundworms occupy all trophic levels in the soil and can be used as environmental indicators (Bongers & Ferris, 1999; Ferris et al., 2001; Yeates et al., 1993; Zhao & Neher, 2014). Nematodes can be categorized into functional groups based on the food resources utilized which include bacterivore, fungivore, omnivore, predator, and plant‐parasitic nematode (PPN) feeding groups (Bongers & Bongers, 1998). Free‐living nematodes (FLN) such as bacterivores, fungivores, omnivores, and predators benefit soil health by contributing to nutrient cycling (Ingham et al., 1985; Schratzberger et al., 2019). Bacterial‐feeding nematodes graze on microbial communities, which can regulate bacterial growth and enhance nutrient mineralization that converts nutrients to forms bioavailable to plants (Ferris & Bongers, 2006; Jiang et al., 2023). Carnivorous nematodes, which include omnivores and predators, prey upon PPN and other invertebrates, thus contributing to top‐down regulation of the soil food web (Wang & McSorley, 2005). Nematodes can be further categorized into colonizer‐persister (cp) groups, based on life history characteristics (Bongers, 1990). Nematodes are categorized on a 1–5 cp group ranging between *r* and *K* strategists. Low cp values are similar to *r* strategists and possess many of the characteristics of colonizer species. They are indicators of resource availability and have a rapid reproduction cycle, short life span, and relatively low sensitivity to environmental disturbance. Persister nematodes, assigned to higher cp values, are most typical of *K* strategists and are associated with food web structure and stability. High cp value nematodes are typically large‐bodied, have long life cycles, and are sensitive to environmental disturbances. Nematode community analyses can be performed based on the nematode abundance and diversity observed to assess the condition of the soil food web. This “snapshot” of the food web condition can be used to evaluate soil health conditions (Ferris et al., 2001).

Turfgrass is an important crop that is impacted by nematodes. PPN can negatively affect turfgrass quality by feeding on turfgrass roots, which can produce patchy areas of declining or dead turfgrass (Crow, 2005). Approximately 8 million hectares of turfgrass are grown in the United States as part of a $40 billion industry (Anonymous, 2017). Many benefits exist for the use of turfgrass as a groundcover and playing surface. Turfgrass aids in water infiltration, reduces erosion, increases property value in residential areas, increases heat dissipation, and reduces injury risk for athletes (Beard & Green, 1994; Loughran et al., 2019). As an esthetic crop, turfgrass often requires fertility inputs and regular thatch maintenance through aerification and topdressing to produce high‐quality appearance and health (Wiecko et al., 1993). Urea and mineral‐based fertilizers are often used to supply nitrogen (N) to turfgrass (Carrow, 1997). However, their use is often scrutinized due to their impacts on the environment. Improperly applied N can leach past the root zone and contaminate groundwater (Petrovic, 1990). To address this concern some states have imposed lawn fertilizer regulations that place limits on when and how much N can be applied to turfgrass (Landschoot et al., 2017). Advances in fertilizer technology have resulted in the development of enhanced efficiency fertilizers (EEFs) that provide season‐long release of N. Because of their low leaching potential, EFFs can be applied at higher single application rates than fertilizers that contain a high portion of quickly available N (Guillard & Kopp, 2004; Hummel & Waddington, 1981; LeMonte et al., 2016; Quiroga‐Garza et al., 2001). The entry of EFFs into the marketplace provides the opportunity to reduce labor and energy inputs into lawn turf management by limiting the number of fertilizer applications to as few as once per year. Organic lawn care programs rely on the use of compost topdressing and other organic materials to supply nutrients to the turf and improve soil health (Guertal & Green, 2012; Johnson et al., 2006). Composted organic sources of N release nutrients over time and aid in pathogen suppression in some situations (Noble & Coventry, 2005). Organic topdressing products are additionally beneficial by recycling nutrients from urban‐generated waste and reducing environmental impacts; however, their use on turfgrass may be restricted in some locations due to the presence of phosphorus (P) in the product (Landschoot et al., 2017).

Nematode community responses to conventional and organic agricultural fertilization and cultivation practices have been studied in continuous cropping and rotations of cereals, forage, and soybean field crops (Li et al., 2018; Liu et al., 2016; Martin et al., 2022; Ugarte et al., 2013). Fertilizer applications often produce short‐term nutrient‐enriched conditions that result in population surges of opportunistic bacterial‐feeding nematodes (Ferris & Bongers, 2006; Gruzdeva et al., 2007). Organic‐based fertilizers and amendments tend to have a stronger overall positive effect on FLN abundance, relative to mineral fertilizers (Bulluck et al., 2002; Ferris & Matute, 2003; Thoden et al., 2011). Nematode community responses to cultivation are often more variable and can affect taxa within a functional group differently (Okada & Harada, 2007). Soil disturbance that inverts subsoil may result in direct effects on nematodes that reduce overall nematode abundance and structure; and potentially lead to conditions that favor *r* strategist populations that are able to recover quickly (Ito et al., 2015; Lenz & Eisenbeis, 2000). Since nematode responses to fertilizer types and cultivation are variable, it is valuable to study specific fertilizer and cultivation combinations for specific cropping systems.

To our knowledge, no studies have been published on long‐term effects of synthetic EEF and organic fertilizer applications and cultivation on nematode communities in tall fescue turfgrass systems. The objective of this study was to investigate the impacts of long‐term fertilization and cultivation practices on nematode community structure and abundance in tall fescue under management for 11 years. Further, the role of soil nutrients on the nematode community composition was evaluated.

## 2 MATERIALS AND METHODS

### 2.1 Study site and plot maintenance

The study was conducted at the University of Maryland Paint Branch Turfgrass Research Facility in College Park, MD (39°00′34.11″N, 76°56′26.33″W). The soil at this site was a Russett (fine‐loamy, mixed semiactive, mesic Aquic Hapludults) and Christiana (fine, kaolinitic, mesic Aquic Hapludults) complex. The top 10 cm of soil consisted of 21% clay, 48% silt, and 31% sand. The study was initiated in October of 2011 on a 4‐year‐old stand of “Titanium” tall fescue and “Raven” Kentucky bluegrass (*Poa pratensis* L.). However, 4 years into the study, because of excessive encroachment of white clover (*Trifolium repens* L.) and rough bluegrass (*Poa trivialis* L.), all vegetation within the study area was killed using quinclorac and glyphosate and the area reseeded at 680 kg ha^−1^ with Firecracker tall fescue. The tall fescue was maintained at 7.5–10 cm with the clippings returned and the turf irrigated as needed to prevent the turf from entering water stress‐induced dormancy. Other than the aforementioned use of quinclorac and glyphosate, pesticide use was limited to a chelated iron product, Fiesta Weed Killer, to control broadleaf weeds. Fiesta was applied on 15 May and 16 June 2021 and on 3 June 2022 at 80 L ha^−1^ in 2250 L ha^−1^ water.

### 2.2 Fertilizer and cultivation treatments

The three fertilizer treatments included a biosolid compost (Orgro, Veolia Water North America Baltimore City Composting Facility, Baltimore, MD), plant‐based yard trimming compost (Leafgro, Maryland Environmental Services, Dickerson, MD), and a synthetic EEF. Cultivation treatments were 0×, 1×, and 2× soil aerification passes. Fertilizer and cultivation treatments were arranged as a randomized complete block design in a 3 × 3 factorial arrangement. From 2011 to 2013 all fertilizer materials were applied at a rate of 156 kg N ha^−1^ once per year between October 3 and 7 with the EFF used having a “marketed” nitrogen release period of 180 days (polymer‐coated urea “Signature” 35‐0‐10, Loveland Products, Inc. Greeley, CO). In 2014, no treatments were applied. In 2015 each treatment was applied twice, once on June 17 and the other on October 13. In all subsequent years, all treatments were applied once per year between October 4 and October 20. In 2015, the single‐use application rate was lowered to 122 kg N ha^−1^ and the EFF changed to a polymer‐coated urea having a 126 to 154‐day nitrogen release rate at 15°C (“Polyon” 42‐0‐0, Harrells, Lakeland, FL). Yearly compost topdressing amounts required to meet the desired N application rate were based on the moist (i.e., as is) bulk density and N content of compost as determined by the University of Massachusetts Soil and Plant Tissue Testing Laboratory (2011–2015), Waypoint Analytical Laboratories (2016–2017) and the Pennsylvania State University Agricultural Analytical Services Laboratory (2018–2021). The P and K content in the two compost materials varied with the year of application, with biosolid compost containing higher levels of P and lower levels of K than yard trimmings compost. Over the course of the entire study the ratio of applied N:P:K was approximately 12:7:1 for biosolids compost and 13:1:4 for yard trimmings compost. The amount of P applied in the two compost materials was above that permissible in some locations where P loading of surface water is a concern (Landschoot et al., 2017). Compost soluble salts and C:N ratios were included in the compost analysis results obtained from the University of Massachusetts and the Pennsylvania State University laboratories. Soluble salts were higher in the biosolid compost (range 4.9–10.3 mmhos cm^−1^) than in the yard trimmings compost (range 1.6–3.3 mmhos cm^−1^). All compost samples had a C:N ratio less than 20:1. Cultivation treatments consisted of 0, 1, or 2 passes of a Ryan GA 30 aerator (Ryan, Div. of Schiller Grounds Care, Inc., Johnson Creek, WI). The aerator was equipped with 1.9 cm by 12.7 cm tines that were spaced 6 cm apart from one another. The cultivation treatments were imposed immediately prior to compost spreading and broadcasting of the EEF. Cores brought to the surface in plots receiving cultivation were left in place.

### 2.3 Soil sampling and data collection

Soil cores were collected on October 18, 2022, prior to the fertilizer application. Samples were collected during the fall to avoid the short‐term effects of cultivation and fertilizer application. Nine 3 cm × 15 cm cores were collected from the center of each plot. Samples were placed in polyethylene sample bags and placed in a cooler during transportation to the lab and stored at 4.5°C prior to extraction. Soil cores from each sample were homogenized and nematodes were extracted from 100 cm^3^ volume of soil by using centrifugal sugar flotation (Jenkins, 1964). Nematodes were fixed in 2% formalin and a subset of 100 nematodes of the total number of nematodes were identified to the genus level using an inverted microscope at 100× magnification (Zeiss, Oberkochen, Germany) according to Bongers (1988), (Goodey, 1963, 2006), and Holovachov et al. (2009). The relative abundance of genera was determined by multiplying the proportion of each genus identified in the 100 nematode subsample by the total number of nematodes in the 100 cm^3^ soil sample. Nematodes were categorized into functional groups based on Yeates et al. (1993) and indicate food source utilization. Nematode genera were additionally assigned to cp groups, broadly reflecting their life history traits (Bongers, 1990).

Soil nematode community diversity and abundance were analyzed by calculating nematode community indices (Bongers, 1990; Ferris et al., 2001; Ferris & Bongers, 2009). Soil food web condition was assessed with the Maturity Index (MI), based on weighted proportions of nematodes present, excluding plant parasites. Maturity Index cp 2‐5 (MI2‐5) was included as a variation of MI that excludes cp‐1 opportunists that may proliferate in response to a soil pollutant (Korthals et al., 1996). Basal Index (BI), Enrichment Index (EI), Structure Index (SI), and Channel Index (CI) were calculated according to Ferris et al. (2001). Visual representation of the soil food web condition was presented with the faunal profile by plotting EI against SI (Ferris et al., 2001).

One‐half of the soil volume from each sample was sent to Waypoint Analytical Virginia, Inc (Richmond, VA) to determine soil organic content through loss on ignition, pH, cation exchange capacity (CEC), and Mehlich 3 extraction levels of P, K, Ca, Mg, S, Fe, Zn, Mn, Cu, Na, and B. Turfgrass quality, percent weed cover, and color (using normalized difference vegetation index: NDVI) data were collected on October 17, 2022. Visual turf quality was based on criteria established by the National Turfgrass Evaluation Program and was rated on a 1–9 scale, where 1 is the bare soil devoid of turf, 6 or above is mostly uniform turf surface of acceptable color, texture, density and 9 is dense, uniform turf possessing optimal turf color. Weed cover was evaluated using the point intersect method (Hoyle et al., 2013). The NDVI of turf was evaluated using a handheld Field Scout CM 1000 NDVI meter (Spectrum Technologies Inc., Aurora, IL) that was directed toward turf at a 45‐degree angle from an approximate height of 1 m.

### 2.4 Statistical analysis

To test the effect of long‐term fertilizer applications and cultivation on nematode counts, we used a generalized linear mixed model (GLMM). A negative binomial distribution was selected to account for overdispersion of the data variance (Brooks et al., 2017; Weaver et al., 2007). Overdispersion is not uncommon from nematode count data due to the aggregated distribution of nematodes in soil (Alake, 2019; Goodell & Ferris, 1980; McSorley, 1982). Fertilizer and cultivation were considered fixed effects and block was considered a random effect. GLMM was calculated using the “glmmTMB” package in R (Brooks et al., 2017; R Core Team, 2021). Pairwise comparisons were conducted with the “emmeans” package (Lenth, 2022). Community indices were calculated using the Nematode Indicator Joint Analysis (NINJA) online web tool (Sieriebriennikov et al., 2014). Nematode index means and turfgrass health measurement (NDVI) means were compared using a two‐way analysis of variance using the “agricolae” R package (de Mendiburu, 2021). Using procedure Rank in SAS software (version 9.4; SAS Institute, Cary, NC) data were ranked. The least‐squares means of treatments were compared using the procedure Mixed in SAS. Significance levels of *α* = 0.001, 0.01, and 0.05 were presented as statistically significant. Non‐metric multidimensional scaling (NMDS) was used to visualize the clustering of soil elements and properties relative to fertilizer and cultivation treatments. The function “vegdist” from the Vegan R package was used to calculate the Bray–Curtis dissimilarity for soil elements and properties (Oksanen et al., 2007). Figures were produced using the ggplot2 package in R (Wickham, 2016).

## 3 RESULTS

### 3.1 Nematodes and ecological indices

Twenty‐two nematode genera were encountered across all soil samples (Table 1). Six genera were within the PPN functional group. Of FLN, eight genera were bacterivores, four genera were fungivores, and four genera were omnivores‐predators. Genera representing less than 1% of total nematodes were not analyzed individually.

**TABLE 1:** Nematode genera identified from tall fescue plots.

Functional group	Genus	Cp^a^ value	Proportion of total nematodes
Plant‐parasites	*Criconemella*	3	0.04
	*Helicotylenchus*	3	0.28
	*Hoplolaimus*	3	0.08
	*Paratylenchus*	2	>0.01
	*Pratylenchus*	3	0.01
	*Xiphinema*	5	>0.01
Bacterivores	*Bunonema*	1	>0.01
	*Cephalobus*	2	0.01
	*Diploscapter*	1	0.03
	*Eucephalobus*	2	0.09
	*Plectus*	2	0.04
	*Prismatolaimus*	3	>0.01
	*Rhabditis*	1	0.03
	*Teratocephalus*	3	>0.01
Fungivores	*Aphelenchoides*	2	0.03
	*Aphelenchus*	2	>0.01
	*Filenchus*	2	0.05
	*Tylenchus*	2	0.31
Omnivores‐Predators	*Aporcelaimellus*	5	>0.01
	*Prodorylaimus*	4	>0.01
	*Ironus*	4	>0.01
	*Mononchus*	4	>0.01

^a^ Cp, Colonizer‐persister value. Cp value is an integer on a 1–5 scale that reflects the life history traits of a nematode genus. Values close to 1 are associated with *r* strategists and values close to 5 are associated with *K* strategists.

Two PPN genera were affected by fertilizer type, with the response of one of the two genera to fertilizer type being dependent on the cultivation intensity (Tables 2 and 3). *Hoplolaimus* counts were significantly greater (*p* ≤ .05) in biosolid‐treated plots than in the synthetic or plant‐based fertilizer‐treated plots (Table 2). *Criconemella* counts were significantly higher (*p* ≤ .05) within the synthetic fertilizer‐treated plots at the 2× cultivation than at the 1× or 0× cultivation (Table 3). The use of either organic fertilizer type resulted in the numerically highest *Criconemella* counts being observed at the 1× cultivation. A fertilizer by cultivation intensity interaction was also observed in the PPN *Helicotylenchus* genus with the numerically highest counts being observed in the synthetic treated plots at 2× cultivation. A similar cultivation response was not observed in *Helicotylenchus* counts for either of the two organic fertilizer types.

**TABLE 2:** Marginal means of nematode genus and functional group counts with no significant interaction (*p* > .05) between fertilizer and cultivation treatments.

Functional group	Genus	Nematode count marginal means/100 cc soil^1^						GLMM summary (*p*‐Value)		
		Fertilizer			Cultivation			Fertilizer	Cultivation	Fertilizer × Cultivation
		Biosolid	Synthetic	Plant‐based	0×	1×	2×			
Plant‐parasite	*Hoplolaimus*	392 a	151 ab	111 b	218	135	224	*	NS	NS
Plant‐parasite	*Pratylenchus*	33	46	14	26	32	27	NS	NS	NS
Bacterivore	*Diploscapter*	40	114	84	77	51	99	NS	NS	NS
Bacterivore	*Eucephalobus*	248 ab	330 a	223 b	297	210	293	*	NS	NS
Bacterivore	*Plectus*	106	190	69	107	90	143	NS	NS	NS
Fungivore	*Filenchus*	121	110	132	143	149	82	NS	NS	NS
Total bacterivores		476 b	694 a	395 b	527 ab	396 b	625 a	***	**	NS
Total fungivores		1075	1334	1173	1175	1191	1201	NS	NS	NS
Total omnivores ‐predators		2 b	5 b	21 a	2 b	14 a	8 a	***	***	NS

*Note*: Main effect marginal means followed by the same letter within a row are not significantly different within fertilizer or cultivation treatments at the .05 probability level.

Abbreviation: NS, not significant.

^1^ Marginal mean values determined by negative binomial generalized mixed model analysis.

*Significant at the .05 probability level. **Significant at the .01 probability level. ***Significant at the .001 probability level.

**TABLE 3:** Marginal means of nematode genus and functional group counts with significant interactions (*p* ≤ .05) between fertilizer and cultivation main effects.

Functional group	Genus	Nematode count marginal means/100 cc soil^1^									GLMM summary (*p*‐value)		
		Biosolid			Synthetic			Plant‐based			Fertilizer	Cultivation	Fertilizer × Cultivation
		0×	1×	2×	0×	1×	2×	0×	1×	2×			
Plant‐parasite	*Criconemella*	55 ab	350 a	11 bc	0 c	0 c	246 a	39 ab	306 a	6 bc	*	NS	***
Plant‐parasite	*Helicotylenchus*	1060 abc	471 c	651 abc	1109 ab	565 bc	1384 a	651 abc	1103 ab	536 bc	NS	NS	*
Bacterivore	*Cephalobus*	4 b	27 ab	6 b	16 b	17 b	126 a	8 b	4 b	25 ab	*	NS	*
Bacterivore	*Rhabditis*	74 bcde	101 abcd	131 abc	156 ab	30 e	204 a	71 bcde	57 cde	43 de	*	*	***
Fungivore	*Aphelenchoides*	55 b	78 ab	92 ab	92 ab	37 b	142 a	88 ab	82 ab	52 b	NS	NS	*
Fungivore	*Tylenchus*	904 ab	788 ab	726 ab	1452 a	775 ab	792 ab	476 b	1214 a	1112 a	NS	NS	*
Total plant‐parasites		1410 ab	1155 abcd	1273 abc	1277 abc	812 cd	1761 a	960 bcd	1440 ab	752 d	NS	NS	***
Total nematodes		3113 abc	2723 bc	2738 abc	3797 ab	2209 c	4206 a	2167 c	3464 abc	2549 bc	NS	NS	**

*Note*: Means followed by the same letter within a row are not significantly different at the .05 probability level.

Abbreviation: NS, not significant.

^1^ Marginal mean values determined by negative binomial generalized mixed model analysis.

*Significant at the .05 probability level. **Significant at the .01 probability level. ***Significant at the .001 probability level.

Three FLN bacterivore genera were affected by fertilizer treatments with the response of two of the genera to fertilizer type being dependent on the cultivation intensity. *Eucephalobus* abundance was significantly greater (*p* ≤ .05) in the synthetic fertilizer‐treated plots than in the biosolid or plant‐based fertilizer plots (Table 2). Plots receiving synthetic fertilizer at the 2× cultivation had the greatest *Rhabditis* and *Cephalobus* genera abundance; with this treatment combination being significantly greater (*p* ≤ .05 and *p* ≤ .001) than 1× cultivation with synthetic fertilizer (Table 3). In contrast, cultivation had no influence on the abundance of *Rhabditis* and *Cephalobus* within the plots receiving the biosolid or plant‐based fertilizer.

No single fertilizer or cultivation treatment effect was observed for any FLN fungivore genera. The *Aphelenchoides* and *Tylenchus* fungivore genera had significant fertilizer by treatment interactions (*p* ≤ .05) however, there were no clear trends in the abundance, as influenced by cultivation, for each three fertilizer materials for the two genera. Total bacterivore abundance was significantly greater (*p* ≤ .01) in 2× cultivation plots than 1× plots and in the plots that received the synthetic fertilizer (*p* ≤ .001). Omnivore‐predator abundance was significantly greater (*p* ≤ .001) in plant‐based fertilizer plots than in biosolid plots (Table 2) and increased with the use of cultivation (*p* ≤ .001). Cultivation affected the nematode total, but only with the use of synthetic fertilizer material (fertilizer by cultivation interaction; *p* ≤ .01, Table 3).

**FIGURE 1:**
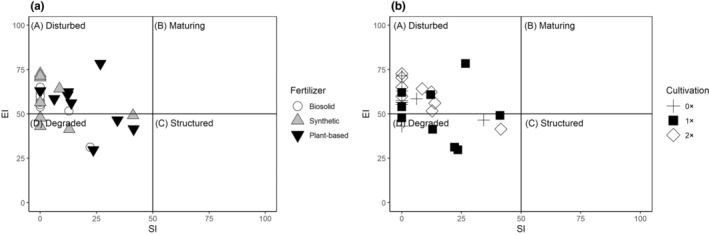
Faunal profile calculated from Enrichment Index (EI) and Structure Index (SI) as affected by fertilizer (a) and cultivation (b) treatments. Data points that fall within quadrat A are associated with a disturbed food web, quadrat B a maturing food web, quadrat C a structured food web, and quadrat D a degraded food web.

Two ecological indices were significantly impacted by long‐term fertilization (Table 4). MI2‐5 was significantly greater (*p* ≤ .05) in plant‐based fertilizer plots than biosolid plots and SI was significantly greater (*p* ≤ .001) in plant‐based plots than synthetic fertilizer and biosolids (Table 4). No indices were significantly affected (*p* > .05) by long‐term cultivation treatments. Distribution of data points in the faunal profile was confined between quadrats A and D (Figure 1).

**TABLE 4:** Nematode community index is affected by fertilizer and cultivation treatments.

Index	Nematode index mean^1^						ANOVA summary (*p*‐Value)		
	Fertilizer treatment			Cultivation treatment			Fertilizer	Cultivation	Fertilizer × cultivation
	Biosolid	Synthetic	Plant‐based	0×	1×	2×			
BI	43.5	40.5	39.1	42.1	44.5	36.6	NS	NS	NS
CI	30.7	23.7	29.3	28.6	34.6	20.6	NS	NS	NS
EI	54.9	57.4	55.2	56.5	50.5	60.5	NS	NS	NS
MI	1.84	1.82	1.91	1.83	1.92	1.81	NS	NS	NS
MI2‐5	2.02 b	2.04 b	2.12 a	2.03	2.09	2.06	*	NS	NS
SI	3.87 b	6.95 ab	18.9 a	4.5	15.4	19.86	*	NS	NS

Abbreviations: BI, Basal Index; CI, Channel Index; EI, Enrichment Index; MI, Maturity Index; MI2‐5, Maturity Index cp 2‐5; NS, non‐significant; SI, Structure Index.

^1^ Means determined with ANOVA.

* Significant at the .05 probability level.

### 3.2 Turf health and soil elements

**FIGURE 2:**
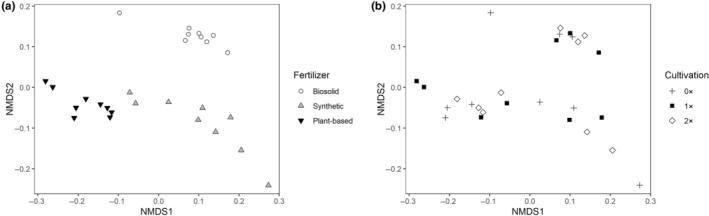
Non‐metric multidimensional scaling ordinations of soil P, K, Mg, Ca, Na, S, Zn Mn, Fe, Cu, B, organic content, CEC, and pH of all 27 soil samples. Fertilizer (a) and cultivation (b) treatments are presented as separate plots with two axes selected for each plot. The stress value was 0.08.

Turfgrass quality ratings, weed counts, and NDVI data were collected (Table 5). NDVI was significantly affected by long‐term fertilizer application. Plant‐based fertilizer plot NDVI was significantly greater (*p* ≤ .05) than biosolid plot NDVI. Quality ratings and number of weeds were not significantly different (*p* > .05) between any treatments. Long‐term cultivation treatments had no significant impact on quality ratings, weed cover, or NDVI (*p* > .05). Significantly different soil element levels (*p* ≤ .05) were measured across fertilizer treatments, but not cultivation treatments (*p* > .05, Table 6). NMDS showed a distinct clustering of soil elements and properties influenced by fertilization, but not cultivation (Figure 2). Biosolid‐treated plots contained the highest levels of Cu, Fe, P, S, and Zn whereas plant‐based fertilizer plots contained the highest concentrations of organic matter, B, K, and Mg and greatest CEC (Table 6).

**TABLE 5:** Turfgrass quality, weed counts, and NDVI measurement means as affected by fertilizer and cultivation treatments taken Oct 17, 2022.

Measurement	Fertilizer			Cultivation			ANOVA summary (*p*‐Value)		
	Biosolid	Synthetic	Plant‐based	0×	1×	2×	Fertilizer	Cultivation	Fertilizer × Cultivation
Quality rating^1^	7.87	8.04	7.90	8.01	7.82	7.98	NS	NS	NS
Weed coverage (%)	7.11	4.89	7.44	6.00	6.78	6.67	NS	NS	NS
NDVI	0.883 b	0.900 ab	0.919 a	0.890	0.912	0.900	*	NS	NS

*Note*: Means within the same row and treatment main effect followed by the same letter are not significantly different.

Abbreviation: NS, non‐significant.

^1^ Quality values in the table represent ranked data on a 1–9 scale with values approaching 9 indicating higher quality. Quality data were analyzed using a mixed model on ranked data.

* Significant at the .05 probability level.

**TABLE 6:** Soil element mean ppm, pH, cation exchange capacity, and organic matter percent from a depth of 0–15 cm following 11 years of fertilizer and cultivation treatments.

Soil parameter	Fertilizer			Cultivation			ANOVA summary (*p*‐Value)		
	Biosolid	Synthetic	Plant‐based	0×	1×	2×	Fertilizer	Cultivation	Fertilizer × cultivation
B^1^	0.39 ab	0.33 b	0.44 a	0.40	0.38	0.39	**	NS	NS
Ca	773 b	723 b	1051 a	858	869	820	***	NS	NS
Cu	2.34	0.74	0.96	1.33	1.33	1.38	***	NS	*
Fe	219 a	124 b	120 b	155	154	153	***	NS	NS
K	136 ab	106 a	166 b	131	135	142	**	NS	NS
Mg	141 b	132 b	170 a	147	150	145	***	NS	NS
Mn	41	49	49	46	45	49	NS	NS	NS
Na	18.6	20.8	18.3	19.8	19.1	18.8	NS	NS	NS
P	138 a	39.1 c	48.1 b	77.3	71.8	76.2	***	NS	NS
S	11.9 a	10.8 ab	9.8 b	11.2	10.6	10.7	***	NS	NS
Zn	5.98 a	1.43 c	2.42 b	3.39	3.12	3.32	***	NS	NS
pH	6.0	5.8	6.0	5.9	5.9	6.0	NS	NS	NS
CEC (meq/100 g)	6.8 b	7.2 b	8.6 a	7.3	7.7	7.6	***	NS	NS
Organic matter (%)	3.43 b	3.43 b	4.12 a	3.77	3.56	3.67	***	NS	NS

*Note*: Means followed by the same letter within a row are not significantly different.

Abbreviation: NS, non‐significant.

^1^ Soil element means presented in ppm.

*Significant at the .05 probability level. **Significant at the .01 probability level. ***Significant at the .001 probability level.

## 4 DISCUSSION

We observed variable impacts on PPN and FLN counts following 11 years of fertilization and cultivation in tall fescue turfgrass. The selected sampling time was chosen to minimize the short‐term effects of annual cultivation and fertilizer applications. All three fertilizer treatments and the 1× and 2× cultivation resulted in significant differences in the abundance of at least one nematode genus or functional group. Notably, counts of an important plant‐parasitic nematode genus, *Hoplolaimus*, were affected by fertilizer treatments. *Hoplolaimus* is one of the most economically important PPN in warm season turfgrasses (Crow, 2021). *Hoplolaimus* densities have previously been shown to be affected by fertilizer source in Southshore creeping bentgrass (Davis & Dernoeden, 2002). *Hoplolaimus* may also have differential sensitivity to compounds that have nematode suppressive activity relative to other PPN. Spikes in *Hoplolaimus* counts following applications of some chemical nematicides that have occurred on golf courses in Florida, adding challenges for management (Crow, 2019, 2021, 2023). In this study, biosolid plot *Hoplolaimus* numbers were near the damage threshold for turfgrass in New England (Wick, 2012) in addition to having the lowest NDVI of the fertilizer treatments. Differential tolerance to environmental contaminants could be partly responsible for the unique density increase in biosolid plots, which no other nematode groups or community indices in this study experienced.

Cultivation impacts on nematode abundance were smaller compared to fertilization. Two functional groups, bacterivores and omnivore‐predators were significantly impacted by cultivation. Nematodes may be physically damaged or moved to less favorable conditions at the soil surface by soil core aeration. Few studies have been devoted to turfgrass cultivation effects on nematodes. White and Dickens (1984) observed low impacts on PPN densities from aeration treatments in bermudagrass. In our study, 2× plots may have experienced sufficient disturbance to favor bacterivore nematode density increases. Omnivores‐predator density was lowest in 0× plots. Omnivore‐predator responses to physical soil disturbance tend to be more variable than responses to heavy metals and nutrient inputs (Zhao & Neher, 2013). Omnivore‐predator nematodes densities are typically unaffected or increase in undisturbed soil compared to cultivated soil (Treonis et al., 2010; Wang et al., 2004; Zhang et al., 2012). However, variability in omnivore‐predator abundance has been previously observed following soil cultivation (Corbett & Webb, 1970; Okada & Harada, 2007; Webb & Corbett, 1973).

Nematode group counts that were affected by statistically significant interactions between treatments lacked consistent patterns with many antagonistic relationships between treatments. Conclusions drawn from these results are highly dependent on the treatment combination and nematode group of interest. *Criconemella* and *Cephalobus* for example, were both consistently numerically more abundant in either 1× or 2× cultivation for each fertilizer treatment. In contrast, *Tylenchus* was numerically most abundant in the 0× treatments for two of the three fertilizer treatments. Further research is needed to elucidate some of the complexities of group‐specific responses from the fertilizer and cultivation interactions observed in this study.

NDVI was lowest in biosolid plots and biosolid plots contained the highest numbers of *Hoplolaimus*. No other PPN genus counts besides *Hoplolaimus* were at northeastern US turfgrass damage thresholds in the study (Wick, 2012). The lower NDVI in biosolid plots might partially be explained by the higher *Hoplolaimus* counts relative to the other treatments. Reduction in PPN counts can positively impact turfgrass NDVI, but additional factors such as environment, nematode species, turfgrass species, and turfgrass stress may influence the relationship between PPN abundance and visual appearance (Groover, 2020; Settle et al., 2007).

FLN abundance and nematode community indices were also affected by turfgrass management. Omnivore predators were largely absent in biosolid plots in our study, only present in two of nine biosolid plots. The presence or absence of functional groups, especially high cp nematode groups like omnivore‐predators, is meaningful in assessing the soil food web condition (Ferris & Bongers, 2009). Previous research has demonstrated the high sensitivity of omnivores predators to environmental contaminants like heavy metals (Georgieva et al., 2002; Korthals et al., 1996; Zullini & Peretti, 1986). Composted biosolids derived from organic sources like industrial and human waste often have relatively high Cu, Fe, and Zn concentrations (Silveira et al., 2003). The greater concentration of Cu, Fe, and Zn observed in biosolid plot soil may have contributed to the very low abundance of omnivore predators in biosolid plots. Notably, turfgrass health was relatively high following 11 years of continuous biosolid applications. Other studies have shown grasses to have tolerance to toxins such as Cd, Cu, Mn, Ni, Pb, and Zn (Gilabel et al., 2014; Gravand et al., 2021; Khan et al., 2018; Shu et al., 2002). Quality ratings in our study indicated no substantial impacts from heavy metals occurred on the appearance of the turfgrass, relative to the other fertilizers.

The low counts of high cp omnivore‐predators nematodes also impacted MI and SI. MI means were not statistically different, but treatment means were near two. MI uses the weighted proportions of the cp groups present in a soil sample to measure if a food web is experiencing disturbed or stable conditions. MI values below two are common for highly managed ecosystems and values above four are associated with undisturbed areas such as forests (Bongers & Ferris, 1999). The low MI2‐5 values indicated low MI values were not due to a high abundance of low cp *r* strategist nematodes proliferating from pollutant‐induced nutrient‐enriched conditions. SI in this study was relatively small, indicating low structure. Greater counts of high cp nematodes were largely responsible for the statistically larger SI in plant‐based fertilizer plots. Lower concentrations of Cu, Fe, and Zn may have reduced the relative impact on high cp omnivore‐predators and therefore, the SI, in plant‐based plots. SI is a measure of food web stability, based on the presence of all cp‐3‐5 nematode represented. SI values approaching zero are associated with a low‐structure environment while values closer to 100 are typical of an undisturbed high‐structure ecosystem (Ferris et al., 2001).

Agroecosystems with frequent or major disturbances may experience conditions that favor opportunistic *r* strategist nematodes (Culman et al., 2010; Lenz & Eisenbeis, 2000). It is common to observe opportunist bacterivore nematode counts increase in response to additions of low C:N ratio fertilizer sources, such as synthetic fertilizer applications (Arancon et al., 2003; Ferris & Bongers, 2006; Gruzdeva et al., 2007). Greater bacterivore abundance in our study was associated with synthetic fertilizer, but it was not correlated with significantly greater nutrient enrichment or greater bacterial‐mediated decomposition according to the EI and CI. The EI values in this study suggest moderate enrichment across fertilizer treatments, with no significant differences. EI is a measure of food web nutrient availability and is calculated as a ratio of opportunistic bacterivore and fungivore nematodes which thrive under enriched conditions, to total enrichment opportunists and basal associated nematodes (Ferris et al., 2001). CI values in our study were most closely associated with bacterial decomposition pathways. CI is used to indicate the dominate decomposition pathway from the proportion of bacterial and fungal feeding nematodes with values closer to 0 indicative of a bacterial decomposition pathway and values closer to 100 associated with a fungal‐dominated decomposition pathway (Ruess & Ferris, 2004). Annual applications of synthetic fertilizer in our study may have provided a sufficiently consistent supply of nutrient forms more favorable to sustained bacterial growth but also resulted in sufficient fungal growth to sustain modest fungivore nematode population density increases.

Faunal profile analysis revealed a disturbed‐degraded environment across fertilizer and cultivation treatments. The soil nematode community characteristics were most closely similar to a disturbed food web. Fertilizer and cultivation treatments resulted in moderately high enrichment and low structure, placing all data points in quadrats A and D, characteristic of a disturbed and degraded environment. Perennial crop systems generally have a structured‐stable faunal profile in quadrats B and C. Our results are more consistent with an annual crop degraded‐disturbed faunal profile (Ferris et al., 2001). Disturbed and degraded food web conditions have been observed in other turfgrass management studies (Cheng et al., 2008; Waldo et al., 2019).

## 5 CONCLUSIONS

The results of our study indicate long‐term fertilization and cultivation practices can impact the densities of plant‐parasitic and free‐living nematodes. Notably, significantly more *Hoplolaimus* and significantly fewer omnivore predators were observed in long‐term biosolid‐treated plots, where Cu, Fe, and Zn concentrations were highest. Turfgrass health was relatively high across all treatments, but biosolid plots had the statistically lowest NDVI relative to plant‐based fertilizer. Additional studies are needed to determine optimal fertilization use and timing to promote free‐living nematode abundance and community structure while minimizing plant‐parasitic nematode abundance to maximize soil and turfgrass health benefits.

## AUTHOR CONTRIBUTIONS

**Benjamin D. Waldo:** Conceptualization (supporting); data curation (equal); formal analysis (lead); investigation (supporting); methodology (equal); resources (supporting); validation (equal); visualization (lead); writing – original draft (lead); writing – review and editing (equal). **Fereshteh Shahoveisi:** Data curation (supporting); formal analysis (equal); investigation (equal); methodology (supporting); resources (equal); supervision (supporting); validation (equal); writing – review and editing (lead). **Mark J. Carroll:** Conceptualization (lead); data curation (equal); funding acquisition (lead); investigation (lead); methodology (lead); project administration (lead); resources (lead); supervision (lead); writing – review and editing (equal).

## CONFLICT OF INTEREST STATEMENT

The authors declare no conflict of interest.

## Supporting information


Appendix S1

Appendix S2

## Data Availability

The data that support the findings of this study are attached as supplemental files with the manuscript submission.

## References

[ece310905-bib-0001] Alake, G. (2019). *Characterization of soil communities using nematode and microbiome analytics* [Doctoral dissertation]. University of Florida, University of Florida Digital Collections. https://original‐ufdc.uflib.ufl.edu/UFE0056062/00001

[ece310905-bib-0002] Anonymous . (2017). *National Turfgrass Federation 2017 The turfgrass industry—Present and future*. National Turfgrass Federation. http://www.turfresearch.org/pdf/Industry%20Turf%20Initiative.pdf.

[ece310905-bib-0003] Arancon, N. Q. , Galvis, P. , Edwards, C. , & Yardim, E. (2003). The trophic diversity of nematode communities in soils treated with vermicompost. Pedobiologia, 47(5–6), 736–740.

[ece310905-bib-0004] Beard, J. B. , & Green, R. L. (1994). The role of turfgrasses in environmental protection and their benefits to humans. Journal of Environmental Quality, 23(3), 452–460.

[ece310905-bib-0005] Bongers, T. (1988). De nematoden van Nederland. Stichting Uitgeverij van de Koninklijke Nederlandse Natuurhistorische Vereniging.

[ece310905-bib-0006] Bongers, T. (1990). The maturity index: An ecological measure of environmental disturbance based on nematode species composition. Oecologia, 83, 14–19. 28313236 10.1007/BF00324627

[ece310905-bib-0007] Bongers, T. , & Bongers, M. (1998). Functional diversity of nematodes. Applied Soil Ecology, 10(3), 239–251.

[ece310905-bib-0008] Bongers, T. , & Ferris, H. (1999). Nematode community structure as a bioindicator in environmental monitoring. Trends in Ecology & Evolution, 14(6), 224–228. 10354624 10.1016/s0169-5347(98)01583-3

[ece310905-bib-0009] Brooks, M. E. , Kristensen, K. , Van Benthem, K. J. , Magnusson, A. , Berg, C. W. , Nielsen, A. , Skaug, H. J. , Machler, M. , & Bolker, B. M. (2017). glmmTMB balances speed and flexibility among packages for zero‐inflated generalized linear mixed modeling. The R Journal, 9, 378–400. 10.3929/ethz-b-000240890

[ece310905-bib-0010] Bulluck, L. R., III , Barker, K. R. , & Ristaino, J. B. (2002). Influences of organic and synthetic soil fertility amendments on nematode trophic groups and community dynamics under tomatoes. Applied Soil Ecology, 21(3), 233–250.

[ece310905-bib-0011] Carrow, R. N. (1997). Turfgrass response to slow‐release nitrogen fertilizers. Agronomy Journal, 89(3), 491–496.

[ece310905-bib-0012] Cheng, Z. , Grewal, P. S. , Stinner, B. R. , Hurto, K. A. , & Hamza, H. B. (2008). Effects of long‐term turfgrass management practices on soil nematode community and nutrient pools. Applied Soil Ecology, 38(2), 174–184.

[ece310905-bib-0013] Corbett, D. C. M. , & Webb, R. M. (1970). Plant and soil nematode population changes in wheat grown continuously in ploughed and in unploughed soil. Annals of Applied Biology, 65(2), 327–335.

[ece310905-bib-0014] Crow, W. T. (2005). Plant‐parasitic nematodes on golf course turf. Outlooks on Pest Management, 16(1), 10–15.

[ece310905-bib-0015] Crow, W. T. (2019). Lance nematodes, an increasing problem on golf courses in the southeastern United States. In Abstracts, Society of Nematologists Annual Meeting.

[ece310905-bib-0016] Crow, W. T. (2021). Diagnosing lance nematodes on bermudagrass. Golf Course Management, 89, 60–64.

[ece310905-bib-0017] Crow, W. T. (2023). Is resistance in plant‐parasitic nematodes a concern with new‐generation nematicides? In Abstracts, Society of Nematologists Annual Meeting.

[ece310905-bib-0018] Culman, S. W. , DuPont, S. T. , Glover, J. D. , Buckley, D. H. , Fick, G. W. , Ferris, H. , & Crews, T. E. (2010). Long‐term impacts of high‐input annual cropping and unfertilized perennial grass production on soil properties and belowground food webs in Kansas, USA. Agriculture, Ecosystems & Environment, 137(1–2), 13–24.

[ece310905-bib-0019] Davis, G. J. , & Dernoeden, P. H. (2002). Dollar spot severity, tissue nitrogen and soil microbial activity in bentgrass as influenced by nitrogen source. Crop Science, 42(2), 480–488.

[ece310905-bib-0020] de Mendiburu, F. (2021). *Agricolae: Statistical procedures for agricultural research*. R package version 1.3‐5. https://CRAN.R‐project.org/package=agricolae

[ece310905-bib-0021] Ferris, H. , & Bongers, T. (2006). Nematode indicators of organic enrichment. Journal of Nematology, 38(1), 3–12. 19259424 PMC2586436

[ece310905-bib-0022] Ferris, H. , Bongers, T. , & De Goede, R. M. (2001). A framework for soil food web diagnostics: Extension of the nematode faunal analysis concept. Applied Soil Ecology, 18(1), 13–29.

[ece310905-bib-0023] Ferris, H. , & Matute, M. M. (2003). Structural and functional succession in the nematode fauna of a soil food web. Applied Soil Ecology, 23, 93–110.

[ece310905-bib-0024] Ferris, H. O. , & Bongers, T. (2009). Indices developed specifically for analysis of nematode assemblages. In M. J. Wilson , & T. Kakouli‐Duarte (Eds.), Nematodes as environmental indicators (pp. 124–145). CABI.

[ece310905-bib-0025] Georgieva, S. S. , McGrath, S. P. , Hooper, D. J. , & Chambers, B. S. (2002). Nematode communities under stress: The long‐term effects of heavy metals in soil treated with sewage sludge. Applied Soil Ecology, 20(1), 27–42.

[ece310905-bib-0026] Gilabel, A. P. , Nogueirol, R. C. , Garbo, A. I. , & Monteiro, F. A. (2014). The role of sulfur in increasing Guinea grass tolerance of copper phytotoxicity. Water, Air, & Soil Pollution, 225, 1–10.

[ece310905-bib-0027] Goodell, P. , & Ferris, H. (1980). Plant‐parasitic nematode distributions in an alfalfa field. Journal of Nematology, 12(2), 136–141. 19300685 PMC2618011

[ece310905-bib-0028] Goodey, J. B. (2006). Freshwater nematodes: Ecology and taxonomy (2nd ed.). Methuen and Co.

[ece310905-bib-1028] Goodey, T. (1963). Soil and freshwater nematodes. Methuen.

[ece310905-bib-1034] Gravand, F. , Rahnavard, A. , & Pour, G. M. (2021). Investigation of vetiver grass capability in phytoremediation of contaminated soils with heavy metals (Pb, Cd, Mn, and Ni). Soil and Sediment Contamination: An International Journal, 30(2), 163–186. 10.1080/15320383.2020.1819959

[ece310905-bib-0029] Groover, W. L. (2020). *Integrated management strategies for plant‐parasitic nematodes on warm‐season turfgrass using plant growth‐promoting rhizobacteria, chemical nematicides, and remote sensing technology* [Doctoral dissertation]. Auburn University. https://etd.auburn.edu/bitstream/handle/10415/7115/Groover%20Dissertation.pdf?sequence=2&isAllowed=y

[ece310905-bib-0030] Gruzdeva, L. I. , Matveeva, E. M. , & Kovalenko, T. E. (2007). Changes in soil nematode communities under the impact of fertilizers. Eurasian Soil Science, 40(6), 681–693.

[ece310905-bib-0031] Guertal, E. A. , & Green, B. D. (2012). Evaluation of organic fertilizer sources for south‐eastern (USA) turfgrass maintenance. Acta Agriculturae Scandinavica, Section B — Soil & Plant Science, 62(Suppl 1), 130–138.

[ece310905-bib-0032] Guillard, K. , & Kopp, K. L. (2004). Nitrogen fertilizer form and associated nitrate leaching from cool‐season lawn turf. Journal of Environmental Quality, 33(5), 1822–1827. 10.2134/jeq2004.1822 15356243

[ece310905-bib-0033] Holovachov, O. , Tandingan De Ley, I. , Mundo‐Ocamp, M. , & De Ley, P. (2009). Identification of Cephaloboidea (Nematoda). Eumaine, Ghent and Nematology, UC Riverside.

[ece310905-bib-1030] Hoyle, J. A. , Yelverton, F. H. , & Gannon, T. W. (2013). Evaluating multiple rating methods utilized in turfgrass weed science. Weed Technology, 27(2), 362–368. 10.1614/wt-d-12-00126.1

[ece310905-bib-0034] Hummel, N. W. , & Waddington, D. V. (1981). Evaluation of slow‐release nitrogen sources on ‘Baron’ Kentucky bluegrass. Soil Science Society of America Journal, 45(5), 966–970. 10.2136/sssaj1981.03615995004500050030x

[ece310905-bib-0035] Ingham, R. E. , Trofymow, J. A. , Ingham, E. R. , & Coleman, D. C. (1985). Interactions of bacteria, fungi, and their nematode grazers: Effects on nutrient cycling and plant growth. Ecological Monographs, 55(1), 119–140.

[ece310905-bib-0036] Ito, T. , Araki, M. , Komatsuzaki, M. , Kaneko, N. , & Ohta, H. (2015). Soil nematode community structure affected by tillage systems and cover crop managements in organic soybean production. Applied Soil Ecology, 86, 137–147.

[ece310905-bib-0037] Jenkins, W. (1964). A rapid centrifugal‐flotation technique for separating nematodes from soil. Plant Disease Reporter, 48(9), 692.

[ece310905-bib-0038] Jiang, Y. , Wang, Z. , Liu, Y. , Han, Y. , Wang, Y. , Wang, Q. , & Liu, T. (2023). Nematodes and their bacterial prey improve phosphorus acquisition by wheat. New Phytologist, 237(3), 974–986. 36285379 10.1111/nph.18569

[ece310905-bib-0039] Johnson, G. A. , Davis, J. G. , Qian, Y. L. , & Doesken, K. C. (2006). Topdressing turf with composted manure improves soil quality and protects water quality. Soil Science Society of America Journal, 70(6), 2114–2121.

[ece310905-bib-0040] Khan, M. M. , Islam, E. , Irem, S. , Akhtar, K. , Ashraf, M. Y. , Iqbal, J. , & Liu, D. (2018). Pb‐induced phytotoxicity in para grass (*Brachiaria mutica*) and Castorbean (*Ricinus communis* L.): Antioxidant and ultrastructural studies. Chemosphere, 200, 257–265. 29494906 10.1016/j.chemosphere.2018.02.101

[ece310905-bib-0041] Korthals, G. W. , van de Ende, A. , van Megen, H. , Lexmond, T. M. , Kammenga, J. E. , & Bongers, T. (1996). Short‐term effects of cadmium, copper, nickel and zinc on soil nematodes from different feeding and life‐history strategy groups. Applied Soil Ecology, 4(2), 107–117.

[ece310905-bib-0042] Landschoot, P. , Carroll, M. J. , Goatley, J. M., Jr. , & Turner, T. R. (2017). Turfgrass nutrient management and regulatory issues in the Chesapeake Bay watershed. International Turfgrass Society Research Journal, 13, 1–11. 10.2134/itsrj2016.05.0419

[ece310905-bib-0043] LeMonte, J. J. , Jolley, V. D. , Summerhays, J. S. , Terry, R. E. , & Hopkins, B. G. (2016). Polymer coated urea in Turfgrass maintains vigor and mitigates nitrogen's environmental impacts. PLoS One, 11, e0146761. 10.1371/journal.pone.0146761 26764908 PMC4713148

[ece310905-bib-0044] Lenth, R. V. (2022). *emmeans: Estimated Marginal Means, aka Least‐Squares Means*. R package version 1.8.1‐1. https://CRAN.R‐project.org/package=emmeans

[ece310905-bib-0045] Lenz, R. , & Eisenbeis, G. (2000). Short‐term effects of different tillage in a sustainable farming system on nematode community structure. Biology and Fertility of Soils, 31, 237–244.

[ece310905-bib-0046] Li, J. , Wang, D. , Fan, W. , He, R. , Yao, Y. , Sun, L. , Zhao, X. , & Wu, J. (2018). Comparative effects of different organic materials on nematode community in continuous soybean monoculture soil. Applied Soil Ecology, 125, 12–17.

[ece310905-bib-0047] Liu, T. , Chen, X. , Hu, F. , Ran, W. , Shen, Q. , Li, H. , & Whalen, J. K. (2016). Carbon‐rich organic fertilizers to increase soil biodiversity: Evidence from a meta‐analysis of nematode communities. Agriculture, Ecosystems & Environment, 232, 199–207.

[ece310905-bib-0048] Loughran, G. J. , Vulpis, C. T. , Murphy, J. P. , Weiner, D. A. , Svoboda, S. J. , Hinton, R. Y. , & Milzman, D. P. (2019). Incidence of knee injuries on artificial turf versus natural grass in National Collegiate Athletic Association American Football: 2004–2005 through 2013–2014 seasons. American Journal of Sports Medicine, 47(6), 1294–1301. 30995074 10.1177/0363546519833925

[ece310905-bib-0049] Martin, T. , Wade, J. , Singh, P. , & Sprunger, C. D. (2022). The integration of nematode communities into the soil biological health framework by factor analysis. Ecological Indicators, 136, 108676.

[ece310905-bib-0050] McSorley, R. (1982). Simulated sampling strategies for nematodes distributed according to a negative binomial model. Journal of Nematology, 14(4), 517–522. 19295746 PMC2618223

[ece310905-bib-0051] Noble, R. , & Coventry, E. (2005). Suppression of soil‐borne plant diseases with composts: A review. Biocontrol Science and Technology, 15(1), 3–20. 10.1080/09583150400015904

[ece310905-bib-0052] Okada, H. , & Harada, H. (2007). Effects of tillage and fertilizer on nematode communities in a Japanese soybean field. Applied Soil Ecology, 35(3), 582–598.

[ece310905-bib-0053] Oksanen, J. , Kindt, R. , Legendre, P. , O'Hara, B. , Stevens, M. H. H. , Oksanen, M. J. , & Suggests, M. (2007). The vegan package. Community Ecology Package, 10, 631–637.

[ece310905-bib-0054] Petrovic, A. M. (1990). The fate of nitrogenous fertilizers applied to turfgrass. Journal Environmental Quality, 19(1), 1–14.

[ece310905-bib-0055] Quiroga‐Garza, H. M. , Picchioni, G. A. , & Remmenga, M. D. (2001). Bermudagrass fertilized with slow‐release nitrogen sources. I nitrogen uptake and potential leaching losses. Journal of Environmental Quality, 30(2), 440–448. 10.2134/jeq2001.302440x 11285904

[ece310905-bib-0056] R Core Team . (2021). R: A language and environment for statistical computing. R Foundation for Statistical Computing. https://www.R‐project.org/

[ece310905-bib-0057] Ruess, L. , & Ferris, H. (2004). Decomposition pathways and successional changes. Nematology Monographs and Perspectives, 2, 547–556.

[ece310905-bib-0058] Schratzberger, M. , Holterman, M. , van Oevelen, D. , & Helder, J. (2019). A worm's world: Ecological flexibility pays off for free‐living nematodes in sediments and soils. Bioscience, 69(11), 867–876. 31719709 10.1093/biosci/biz086 PMC6829015

[ece310905-bib-0059] Settle, D. M. , Fry, J. D. , Milliken, G. A. , Tisserat, N. A. , & Todd, T. C. (2007). Quantifying the effects of lance nematode parasitism in creeping bentgrass. Plant Disease, 91(9), 1170–1179. 30780659 10.1094/PDIS-91-9-1170

[ece310905-bib-0060] Shu, W. S. , Ye, Z. H. , Lan, C. Y. , Zhang, Z. Q. , & Wong, M. H. (2002). Lead, zinc and copper accumulation and tolerance in populations of *Paspalum distichum* and *Cynodon dactylon* . Environmental Pollution, 120(2), 445–453. 12395858 10.1016/s0269-7491(02)00110-0

[ece310905-bib-0061] Sieriebriennikov, B. , Ferris, H. , & de Goede, R. G. (2014). NINJA: An automated calculation system for nematode‐based biological monitoring. European Journal of Soil Biology, 61, 90–93.

[ece310905-bib-0062] Silveira, M. L. A. , Alleoni, L. R. F. , & Guilherme, L. R. G. (2003). Biosolids and heavy metals in soils. Scientia Agricola, 60(9), 793–806.

[ece310905-bib-0063] Thoden, T. C. , Korthals, G. W. , & Termorshuizen, A. J. (2011). Organic amendments and their influences on plant‐parasitic and free‐living nematodes: A promising method for nematode management? Nematology, 13(2), 133–153.

[ece310905-bib-0064] Treonis, A. M. , Austin, E. E. , Buyer, J. S. , Maul, J. E. , Spicer, L. , & Zasada, I. A. (2010). Effects of organic amendment and tillage on soil microorganisms and microfauna. Applied Soil Ecology, 46(1), 103–110.

[ece310905-bib-0065] Ugarte, C. M. , Zaborski, E. R. , & Wander, M. M. (2013). Nematode indicators as integrative measures of soil condition in organic cropping systems. Soil Biology and Biochemistry, 64, 103–113.

[ece310905-bib-0066] Waldo, B. D. , Grabau, Z. J. , Mengistu, T. M. , & Crow, W. T. (2019). Nematicide effects on non‐target nematodes in bermudagrass. Journal of Nematology, 51(1), 1–12. 10.21307/jofnem-2019-009 PMC6929642 31088021

[ece310905-bib-0067] Wang, K. H. , McSoreley, R. , & Gallaher, R. N. (2004). Relationship of soil management history and nutrient status to nematode community structure. Nematropica, 34(1), 83–95.

[ece310905-bib-0068] Wang, K. H. , & McSorley, R. (2005). *Effects of soil ecosystem management on nematode pests, nutrient cycling*, *and plant health*. APSnet Feature Articles. 10.1094/APSnetFeatures/2005-0105

[ece310905-bib-0069] Weaver, D. B. , Lawrence, K. S. , & van Santen, E. (2007). Reniform nematode resistance in upland cotton germplasm. Crop Science, 47(1), 19–24.

[ece310905-bib-0070] Webb, R. M. , & Corbett, D. C. M. (1973). The effect of phorate on nematode populations in wheat grown continuously on ploughed and unploughed soil. Soil Biology and Biochemistry, 5(5), 585–591.

[ece310905-bib-1069] White, R. H. , & Dickens, R. (1984). Plant‐parasitic nematode populations in bermudagrass as influenced by cultural practices 1. Agronomy Journal, 76(1), 41–43.

[ece310905-bib-0071] Wick, R. (2012). *Nematodes on golf greens*. University of Massachusetts Extension. https://ag.umass.edu/turf/fact‐sheets/nematodes‐on‐golf‐greens

[ece310905-bib-0072] Wickham, H. (2016). ggplot2: Elegant graphics for data analysis. Springer‐Verlag.

[ece310905-bib-0073] Wiecko, G. , Carrow, R. N. , & Karnok, K. J. (1993). Turfgrass cultivation methods: Influence on soil physical, root/shoot, and water relationships. International Turfgrass Society Research Journal, 7, 451–457.

[ece310905-bib-0074] Yeates, G. W. , Bongers, T. , De Goede, R. G. , Freckman, D. W. , & Georgieva, S. (1993). Feeding habits in soil nematode families and genera—An outline for soil ecologists. Journal of Nematology, 25(3), 315–331. 19279775 PMC2619405

[ece310905-bib-0075] Zhang, X. , Qi, L. , Zhu, A. , Liang, W. , Zhang, J. , & Steinberger, Y. (2012). Effects of tillage and reside management on soil nematode communities in North China. Ecological Indicators, 13(1), 75–81.

[ece310905-bib-0076] Zhao, J. , & Neher, D. A. (2013). Soil nematode genera that predict specific types of disturbance. Applied Soil Ecology, 64, 135–141.

[ece310905-bib-0077] Zhao, J. , & Neher, D. A. (2014). Soil energy pathways of different ecosystems using nematode trophic group analysis: A meta analysis. Nematology, 16(4), 379–385.

[ece310905-bib-0078] Zullini, A. , & Peretti, E. (1986). Lead pollution and moss‐inhabiting nematodes of an industrial area. Water, Air, and Soil Pollution, 27, 403–410.

